# Cardiac Resynchronization Therapy and Left Atrial Remodeling: A Novel Insight?

**DOI:** 10.3390/biomedicines11041156

**Published:** 2023-04-12

**Authors:** Andrei Dan Radu, Alexandru Zlibut, Alina Scarlatescu, Cosmin Cojocaru, Stefan Bogdan, Alexandru Scafa-Udriște, Maria Dorobantu

**Affiliations:** 1 Faculty of Medicine, “Carol Davila” University of Medicine and Pharmacy, 050474 Bucharest, Romania; radu_dan_andrei@yahoo.com (A.D.R.); alina.scarlatescu@gmail.com (A.S.); cojocaru.r.b.cosmin@gmail.com (C.C.); stefan_n_bogdan@yahoo.com (S.B.); alexscafa@yahoo.com (A.S.-U.); mariadorobantu@yahoo.com (M.D.); 2 Cardiology Department, Emergency Clinical Hospital of Bucharest, 014461 Bucharest, Romania; 3 Department of Internal Medicine, Iuliu Hatieganu University of Medicine and Pharmacy, 400012 Cluj-Napoca, Romania; 4 Cardiology Department, Elias University Emergency Hospital, 011461 Bucharest, Romania

**Keywords:** cardiac resynchronization therapy, left atrial volume, left atrial strain, left atrial remodeling, left atrial phasic function

## Abstract

Cardiac resynchronization therapy (CRT) restores ventricular dyssynchrony, improving left ventricle (LV) systolic function, symptoms, and outcome in patients with heart failure, systolic dysfunction, and prolonged QRS interval. The left atrium (LA) plays tremendous roles in maintaining cardiac function, being often inflicted in various cardiovascular diseases. LA remodeling implies structural—dilation, functional—altered phasic functions, and strain and electrical—atrial fibrillation remodeling. Until now, several important studies have approached the relationship between LA and CRT. LA volumes can predict responsiveness to CRT, being also associated with improved outcome in these patients. LA function and strain parameters have been shown to improve after CRT, especially in those who were positive responders to it. Further studies still need to be conducted to comprehensively characterize the impact of CRT on LA phasic function and strain, and, also, in conjunction with its impact on functional mitral regurgitation and LV diastolic dysfunction. The aim of this review was to provide an overview of current available data regarding the relation between CRT and LA remodeling.

## 1. Background

Cardiac resynchronization therapy (CRT) has a tremendous impact on relieving symptoms, and improving prognosis and quality of life, in patients with heart failure (HF) with decreased left ventricle (LV) ejection fraction (LVEF) and prolonged QRS interval, especially when left bundle branch (LBB) block is present. Nonetheless, there are still important gaps in identifying proper candidates for CRT and in accurately predicting major adverse cardiovascular events (MACEs). Thus, many risk score systems have been deployed. However, due to lack of cross-validation and small numbers of patients, they are still not able to be used in day-to-day practice [1].

Until now, the research trend has been towards developing imaging methods that might predict CRT responsiveness and outcome. However, they mainly focused on the functioning of the LV. Standard two-dimensional (2D) echocardiography has been used to determine various imaging parameters which might predict the efficacy of CRT. Amongst all, the presence of septal flash and apical rocking were the most relevant echocardiography-based parameters that correlated with CRT response rate and improvement in functional mitral regurgitation, while others provided questionable results [2]. A recently published article has proved the utility of interventricular septum’s viability and LV work asymmetry determined by cardiac magnetic resonance imaging (CMR) as future accurate predictors to identify CRT responders [3].

More and more attention is being given to the left atrium (LA). Due to continuous technological development, the paramount implications of LA in atrial fibrillation, ischemic heart disease (IHD), and even in HF have been proved. Nonetheless, in patients with dyssynchrony who might benefit from CRT, the role of LA has not been yet fully elucidated. LA remodeling determined using standard two-dimensional (2D) echocardiography independently predicted cardiovascular outcome in patients with heart failure (HF), while lower LA peak longitudinal strain was closely related to a higher incidence of atrial fibrillation [4].

The aim of this review was to provide an overview of current available data regarding the relation between CRT and LA remodeling.

## 2. LA Remodeling: From Physiology to Pathophysiology

LA plays important roles in the cardiac cycle, ensuring its continuity by acting as a diastolic pump for the LV. LA function can be systematized as three-phased phenomena comprising reservoir, conduit, and active booster pump functions [5]. Initially, the LA functioning begins when the oxygenated blood that returns through the pulmonary veins is stored in the LA in the course of LV systole and isovolumetric relaxation. Afterwards, most of the blood which is stored in the LA inertly passes into the LV, while in the end of the diastole, atrial contraction occurs and up to 30% of the stroke volume is then transferred into the LV [6,7,8,9].

Being a dynamic process, there are several hemodynamic and neuro-endocrine factors which significantly influence the physiology of LA function [5]. LA preload is majorly based on the blood volume, whereas LV stiffness and biomechanics are the main factors that influence LA afterload. Thus, LV diastolic dysfunction and increased filling pressures are significant determinants for LA dysfunction [8,10,11]. To some extent, LA physiological increase in LV volume and pressure determines a more efficient LA compliance. Nevertheless, LA enlargement beyond normal ranges quickly impairs atrial function [12,13]. There are also other clinical factors that influence the functioning of the LA. Aging, especially after 65 years, usually leads to LV stiffness, decreased distensibility, and impaired pressure–volume curves [13]. This mainly leads to increased left atrial active booster pump function to ensure efficient LV filling [14]. As for athletes, they have notably increased LV stroke volume and, secondary to this, LA passive and active emptying volumes are also higher [15]. On the other hand, the impact of neuro-endocrine humoral factors is of great significance on atrial function by influencing heart rate, blood pressure, and cardiac output [5]. Moreover, besides blood volume, the LA’s physiology is also impacted by the intracavitary pressures secondary to blood fillings. Thus, the atrial Frank–Starling phenomena ensure myocardial fibers’ stretching and contractility force by modulating calcium affinity. Nonetheless, it has been shown that sensitizing atrial myocardial fibers to calcium might increase the arrhythmogenic risk for atrial fibrillation [16].

Akin to LV remodeling, LA remodeling encompasses anatomical, functional, and electrophysiological alterations within the atrium which are closely related to cardiovascular disease [17]. It implies maladaptation within LA dimension and shape, along with function and electrophysiological abnormalities [18]. Due to its thin structure, the first and foremost notable change within the atrium is LA dilation, having various pathological significances: in apparently healthy individuals, as cardiovascular risk factor, in those with atrial fibrillation, mitral valve diseases or HF, as a red flag of disease progression. Furthermore, LA mechanical remodeling, which includes alterations within all three phasic functions, is more significantly associated with cardiovascular illnesses and, sometimes, might occur even before LA’s enlargement [19]. Additionally, LA rigidity and impaired relaxing significantly influence all three LA functions, being involved in cardiovascular illnesses such as HF, IHD, and mitral regurgitation [20].

The pathogenetic backbone of LA remodeling implies a complex mismatch in terms of energy source change, increased natriuretic peptides, and pro-inflammatory molecules (angiotensin II, transforming growth factor-beta, aldosterone, C-reactive protein, interleukins, and various cytokines) [21,22,23,24]. These molecular phenomena lead to atrial structural disarray, increased tissue fibrosis, electrical inhomogeneity, and global dysfunction. Atrial interstitial fibrosis and dilation are markers for LA morphological remodeling, and altered parameters of LA functioning are hallmarks for LA functional remodeling, while atrial tachyarrhythmias suggest LA electrophysiological remodeling [25].

## 3. The Contemporary Role of CRT in Modern Cardiology

### 3.1. Technical Aspects of CRT

CRT is the most advanced method of cardiac pacing and implies the presence of three leads: one in the right atrium (for patients in sinus rhythm, while for those who have atrial tachyarrhythmias this lead is useless), another in the right ventricle (for right ventricle pacing), and the other one in the coronary sinus (for left ventricular pacing). The main purpose of the LV lead is to approach the myocardial location with the latest intrinsic activation; thus, this lead is usually placed towards the lateral or postero-lateral myocardial wall of the LV, far from the right ventricle lead. An important aspect is also to avoid the fibrotic areas of the myocardium in order to obtain optimal biventricular pacing and restore synchronicity [26].

Placing the lead into the coronary sinus and establishing its final location many times represent important drawbacks which impact the efficacy of LV pacing, especially due to anatomical variants of the heart’s veins, lack of lead stability, and phrenic nerve stimulation [27]. Nonetheless, approaching the LV myocardial area with the latest intrinsic activation is of paramount importance for the final result in tackling cardiac dyssynchrony and to improve cardiac outcome [28,29,30], but this is not always possible due to abnormal cardiac veins’ anatomy or the presence of fibrosis [31].

Nonetheless, regarding quadripolar lead-based vendors, it has been shown that these types of leads determine tremendous electrical responses, thus improving HF and resynchronization therapy [32].

### 3.2. Current Indications for CRT

Various clinical trials and studies have confirmed the current position of CRT in managing and treating patients with ischemic heart disease and non-ischemic dilated cardiomyopathy and LV systolic dysfunction. Emerging data endorse the tremendous impact of CRT on increasing survival and reducing MACEs in this category of patients by improving LV function and halting and even reversing LV remodeling [33].

Actually, current international guidelines recommend CRT in patients with HF with a reduced LV systolic function (LVEF of under 35%) who are symptomatic in spite of optimal medical treatment and have a duration of QRS interval of over 130 ms with an LBB block morphology. The category of patients with LV systolic dysfunction who receive the maximum level and class of scientific evidence is that of patients with a QRS duration of at least 150 ms and an LBB block aspect, who do not have supraventricular tachyarrhythmias, while in those with a QRS interval duration between 130 and 149 ms, the level of recommendation decreases by a class and level [34,35,36]. However, if the QRS interval is narrow, no clinical trial has proved any improvement in symptoms, NYHA class, or outcome, despite any other positive clinical or imaging criteria [37,38]. On the contrary, recently reported papers have concluded that, in the absence of dyssynchrony, CRT determines itself by its functioning and dyssynchrony, thus negatively impacting the prognosis of such patients [39]. In this idea, the trials LESSER-EARTH and ECHO-CRT, which aimed to evaluate if patients with cardiovascular disease who present with narrow QRS interval would benefit from CRT, were briefly terminated due to their detrimental impact on morbidity and survival of these subjects [40,41].

CRT aims to restore the electrical dyssynchrony that is mainly determined by the presence of prolonged QRS interval, especially when the LBB is blocked. This electrical malfunctioning is mostly responsible for promoting LV remodeling and both systolic and diastolic dysfunctions [42]. One of the most important aspects in the proper selection of patients who would benefit from CRT is the presence of an LBB block, which can be identified using conventional electrocardiogram. Nevertheless, when it comes to interpreting whether one has a typical LBB block, there is major unevenness caused by a dearth of appropriate definition of it, which, in turn, leads to significant variability between different observers [43]. Interestingly, a meta-analysis supports the independent ability of QRS interval to predict all-cause mortality, beyond LV systolic dysfunction and QRS morphology [44]. However, patients with a non-LBB block aspect of the QRS have been found to have rather unpredictable therapeutic response rates, which also varied with the type of their cardiac disease [45].

Last but not least, for patients with a right bundle branch (RBB) block, beneficial effects have been seen in those with HF and reduced LVEF of under 35% and a widened QRS complex of over 150 ms, while in those with a duration of QRS between 130 and 149, the benefit was questionable. Moreover, in those with a typical RBB block and QRS complex of under 150 ms, data do not support the efficacy of CRT [46].

### 3.3. Evaluating the Efficacy of CRT

In a joint-position paper by the Heart Failure Association, European Heart Rhythm Association, and European Association of Cardiovascular Imaging, they stated that most patients who might have benefited from CRT in terms of life quality, HF hospitalization, and survival, did not benefit from it. Furthermore, they reported also non-efficient follow-up and monitoring, which also halted the impact of CRT. To tackle these shortcomings, they proposed improvements relating to CRT underutilization, correctly establishing patients’ characteristics, renouncing the term “non-responsive”, especially because HF is a progressive illness and even delaying or stopping its progression should be considered a positive clinical response to CRT, and close post-implantation monitoring [47]. Furthermore, the RESERSE study aimed to properly characterize the impact of CRT on survival and to provide a new reclassification accordingly. They showed that patients who presented with disease progression under CRT at follow-up had increased risk of death, while those who early stabilized or clinically improved under CRT had significantly increased survival. Thus, the research team proposed a three-way reclassification, comprising Worsened, Stabilized, and Improved [48]. Moreover, in the study by Nakai et al. conducted in patients who underwent CRT and were followed-up at 6 months, they aimed to assess both functional responses, defined as improvement in NYHA class with at least one category, and echocardiographic response, viewed as a lower LV end-systolic volume with at least 15% or improvement in LVEF with at least 5%. They concluded that functional response was associated with an improved response rate and outcome, as compared to the echocardiographic response [49].

The main studies endorsing the utility of CRT in patients with HF are presented in Table 1 [40,50,51,52,53,54,55,56,57,58].

As cardiovascular imaging constantly advances, imaging-based methods in guiding CRT have been recently proposed. In the study by Kheiri et al., they sought to evaluate if imaging-guided LV lead placement might have beneficial impacts. They found that imaging-guided LV lead placement significantly improved clinical, echocardiographic, and outcome status, even in those without non-LBB block morphology of the QRS [59]. Moreover, another recently published study aimed to assess the role of multimodal imaging in increasing response rates to CRT. Echocardiography-based strain was used to identify the myocardial region with the latest intrinsic activation. Cardiac computed tomography was used to properly describe the venous anatomy of the heart and to properly select venous branches where the LV lead was to be positioned. CMR was used to characterize myocardial tissue, especially from the region of the latest mechanical activation. Nonetheless, combined multimodal imaging failed to provide any beneficial impact in terms of CRT positive response or in predicting cardiac death or HF hospitalization [60].

## 4. Assessment of LA Function by Cardiac Functional Imaging

### 4.1. Echocardiography

Echocardiography is the main imaging tool used for assessing LA anatomy and function. All three phasic functions can be evaluated using standard 2D echocardiography, being an accurate and reproducible technique [5]. The most useful and easily assessed parameter of LA remodeling is maximum LA volume, which is best assessed using Simpson’s biplane method [61]. In the study by Bhat et al. conducted on healthy volunteers, they sought to assess hemodynamic changes in LA function using echocardiography. They found that both maximum and minimum LA volumes decreased at exertion, thus decreasing the LA reservoir fraction, while increasing the LA conduit fraction [62]. Maximum LA volume has been shown to be significantly associated with MACEs [63]. However, increasing evidence suggests the utility of minimum LA volume in predicting increased LV filling pressures and diastolic dysfunction, being also considerably associated with impaired pulmonary wedge pressure at heart catheterization [64,65]. In the study by Thadani et al., they showed that minimum LA volume was a strong predictor for HF hospitalization, myocardial infarction, stroke, and all-cause mortality in patients with chronic coronary syndromes [66]. Nevertheless, 3D echocardiography has been shown to provide superior data regarding both minimum and maximum LA volumes. In the study by Wu et al. that sought to evaluate the predictive ability of minimum LA volume determined by 3D echocardiography, it was shown that, even though both maximum and minimum LA volumes significantly predicted MACEs at 2.5-year follow-up, minimum LA volume notably ensured a superior incremental predictive value over maximum LA volume [67]. Furthermore, the study by Russo et al. followed to assess the prognosis ability of LA volumes and reservoir function using 3D echocardiography in elderly subjects. They proved that 3D echocardiography-based LA volumes significantly predicted MACEs, while minimum LA volume was superior to other parameters and performed best [65]. Similarly, another study showed the superiority of 3D echocardiography and especially of minimum LA volume in predicting cardiac death, stroke, and myocardial infarction [68].

Furthermore, using maximum, minimum, and pre-atrial contraction LA volumes, all three phasic functions can be determined, resulting in LA total emptying volume (LATEV) and fraction (LATF), LA passive emptying volume (LAPEV) and fraction (LAPF), and LA active booster pump emptying volume (LAAEV) and fraction (LAAF) [61]. Recently, in order to establish homogeneity between measurements of LA phasic functions, the European Association of Cardiovascular Imaging, within the NORRE study, reported specific values for these parameters within healthy volunteers. Thus, for reservoir function: LATF of 68.5% (IQR: 63.2–73.2), LAPF of 43% (IQR: ±10.2), and LAAF of 43.1% (IQR: ±9.4). They also showed that these values significantly increase with age, especially in those over 60 years [69].

Another category of echocardiography-based measurements for assessing atrial remodeling is LA strain and strain rates. The most useful echocardiography method is represented by speckle-tracking imaging for determining LA reservoir strain (LAS-r), LA conduit strain (LAS-c), and LA active strain (LAS-a) [61]. Although strain is a promising tool in predicting LA remodeling, several technical limitations due to LA anatomy and heterogeneous thickness must always be considered [70]. Nevertheless, increasing evidence endorses the utility of LA strains in various cardiovascular diseases. In patients with persistent atrial fibrillation, LAS-r was closely associated with the presence of LA appendage thrombus [71], while impaired LAS-a was significantly correlated with the occurrence of atrial fibrillation, its progression from paroxysmal to persistent, and also with stroke [72,73]. In patients with HF with reduced LVEF, alterations in LAS-r were associated with atrial and ventricular enlargements, impaired LV global longitudinal strain and LVEF, right ventricular dysfunction, and increased LV filling pressures. Furthermore, LAS-r proved to be a useful predictor for MACEs in this category of patients [74]. Moreover, LAS-r was also correlated with cardiac death, atrial fibrillation, serum levels of natriuretic peptides, and NYHA class [4,75].

### 4.2. CMR

Emerging data endorse the clinical utility of CMR-derived LA volumes and function parameters in assessing risk stratification and prognosis prediction in patients with various cardiovascular diseases. CMR can be used for assessing LA remodeling, being also superior in terms of tissue characterization. It can accurately assess LA size and volumes, while all three LA phasic functions can also be approached using this method. With several limitations regarding wall thickness and complex anatomy, CMR can assess the presence and extent of myocardial fibrosis within LA [61]. Recently, in the study by Li et al., the authors sought to determine physiological reference values for LA phasic function parameters. As compared to short-axis measurements of the LA, the biplane method was accompanied by biases in range values. Although the absolute values for LA volumes had significant gender differences, after indexing for body-mass surface, the variances were almost inexistent. As for LA phasic functions, there were not significant differences between the two genders [76].

A study conducted on subjects from the MESA study group sought to evaluate the ability of LA volumes and function determined by CMR to predict the occurrence of atrial fibrillation. Individuals who developed atrial fibrillation had significantly higher LA volumes and impairment in all three LA phasic functions. Furthermore, the peak longitudinal LA strain was impaired. After adjustment for covariates, maximum LA volume, peak longitudinal LA strain, LATF, and LAPF were independent predictors for atrial fibrillation [77]. Moreover, in the study by Khan et al. conducted on subjects without clinically overt cardiovascular diseases, the authors sought to evaluate the impact of LA enlargement on all-cause mortality. They found that dilated LA was significantly associated with aging, atrial fibrillation, arterial hypertension, HF, and LV dilation. Furthermore, moderate and severe enlargement in LA were independently associated with all-cause mortality [78].

Moreover, regarding LA phasic functions, more and more studies have provided valuable information regarding the utility of CMR in determining these parameters. In the study by Chirinos et al., which compared patients without HF with those with HF with preserved LVEF and reduced LVEF, significant differences were shown in terms of LA phasic functions between groups. Those with HF and reduced LVEF had significantly impaired LA phasic functions as compared to the other two groups. Interestingly, these findings persisted even after adjustment for age, gender, body-mass index, arterial hypertension, diabetes mellitus, or IHD [79]. Moreover, in a study conducted on patients with different degrees of diastolic dysfunction, it was shown that LA volumes, and impaired LATF, LAS-r, and LAS-a were significantly associated with the severity of diastolic dysfunction [80]. Similarly, another study proved that LA phasic function determined by CMR might provide important subclinical information regarding LA remodeling in patients who develop paroxysmal atrial fibrillation [81].

Furthermore, CMR is able to efficiently determine LA strains with increasing evidence in providing accurate prognostic prediction. In the study by Kowallick et al., they sought to evaluate the efficacy and reproducibility of CMR in appraising LA strains and strain rates. They showed that CMR is able to accurately assess LA longitudinal strain and strain rates using standard cine-CMR images [82]. Similarly, a recently published paper sought to assess the normal values of LA strains by CMR and to compare them to those determined using standard 2D echocardiography in healthy volunteers. They found that, not only were the parameters accurately comparable to those determined by echocardiography, but they also proved that LAAF, LAAEV, LAS-r, and LAS-a rate significantly increased with advanced aging, while LAS-c considerably decreased with it [83]. In the study by Raafs et al. conducted in patients with dilated cardiomyopathy, LAS-c by CMR was an independent predictor for sudden cardiac death, tachyarrhythmias, and HF hospitalization, and was superior to impaired LV global longitudinal strain and decreased LVEF, and added incremental value to late gadolinium enhancement. Furthermore, LAS-a independently predicted the occurrence of atrial fibrillation [84]. In the study by Nayyar et al., which followed the occurrence of MACEs after acute myocardial infarction, it was shown that LAS-r by CMR was an independent predictor for outcome, even after adjustment for confounders [85]. Moreover, in an interesting study conducted on patients following catheter ablation for atrial fibrillation and which aimed to assess the ability of LA parameters to predict arrhythmia’s recurrence, it was shown that lower LAS-a significantly predicted the relapse of atrial fibrillation after catheter ablation [86].

## 5. The Impact of CRT on LA Remodeling Evaluated by Cardiac Functional Imaging

### 5.1. LA Volume

**Figure 1:**
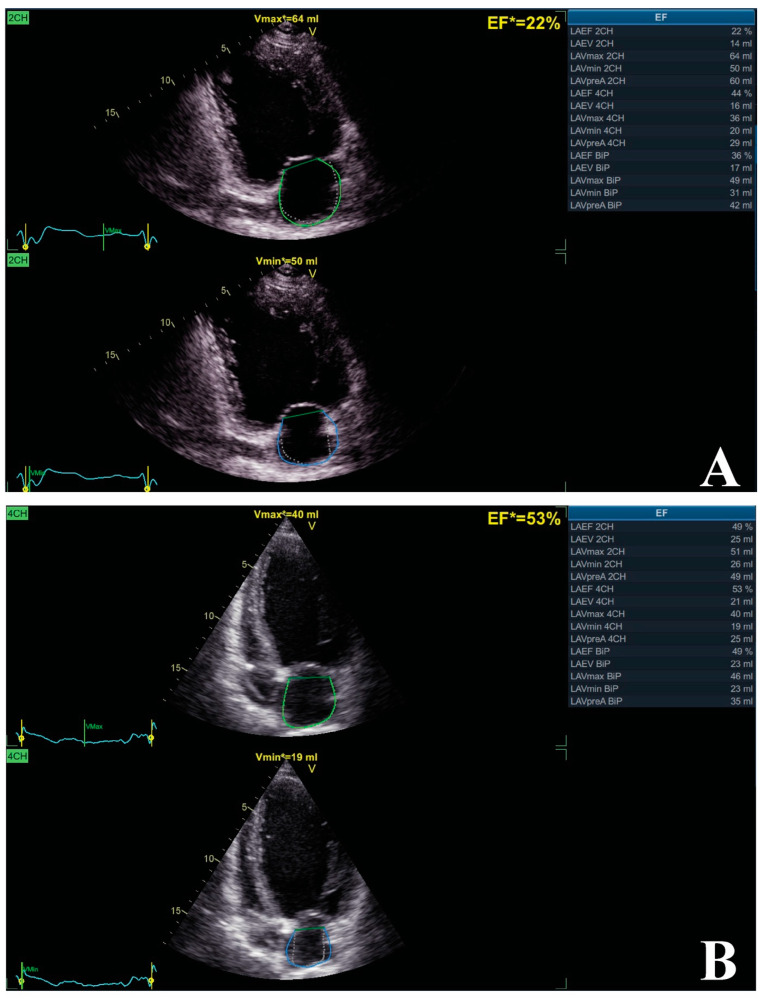
The impact of CRT on LA volumes. Patient with dilated cardiomyopathy, LVEF of 33%, and QRS morphology of LBBB with duration of 153 msec. Echocardiographic evaluation using a General Electric VIVID E95 vendor and EchoPac software package to determine LA biplane volumes (Images from the personal collection of the authors). Initial evaluation showed increased LA volumes (**A**) and, after a 6-month follow-up, significant LA remodeling with reduction in LA volumes can be seen (**B**). Abbreviations: LA, left atrium; LBBB, left bundle branch block; LVEF, left ventricle ejection fraction.

Emerging evidence suggests the positive impact of CRT on LA remodeling parameters, especially on LA volumes, which significantly improve as much as the heart responds to the reestablishment of synchrony. Kuperstein et al. evaluated the impact of CRT on LA volumes in patients from the MADIT–CRT trial. Initially, they proved that patients with higher values of LA volumes had increased risk of all-cause mortality, HF, and cardiac death, while CRT had significant impact in promoting LA reverse remodeling. Furthermore, they showed that CRT was associated with significant decrease in LA volume and, also, they found that every lowering in LA volume by 1% was associated with 4% reduction in the hazard ratio of HF and death [87]. Furthermore, another interesting study, conducted in patients from the MADIT–CRT study, sought to evaluate if the reduction in LA volumes secondary to CRT might reduce the risk of atrial tachyarrhythmias. They found that decreased LA volumes in those who were also responders to CRT had significantly lower risk of developing atrial tachyarrhythmias and had reduced risk of outcome [55]. Figure 1 represents an example of LA reverse remodeling after CRT.

A recently published meta-analysis sought to evaluate the relationship between CRT and LA volumes and included 2191 patients from 10 studies. It concluded that CRT responders had lower LA volumes at baseline, while they decreased after CRT implantation. Furthermore, it found that a baseline indexed maximum LA volume of under 34 mL/m^2^ predicted positive response to CRT [88]. Moreover, in the study by Badran et al., they aimed to assess the interrelation between LA function and dimensions and CRT. They found that CRT responders had significant reduction in both maximal and minimal LA volumes, while atrial fibrillation was more frequent in the CRT non-responders’ group [89]. Likewise, Rossi et al. aimed to evaluate the ability of LA volumes in predicting the responsiveness to CRT. Maximum LA volume was independently associated with CRT positive response at follow-up [90]. Another similar study also found that maximum LA volume was lower in CRT responders. However, they also showed that there were no differences for LA volume in terms of the time difference between QRS onset and the end of LV ejection, nor between QRS duration of over 150 ms and above this value [91]. Similarly, another study reported improvement in both maximum and minimum LA volumes early after CRT implantation, being also associated with diastolic filling time/RR interval ratio [92].

### 5.2. LA Phasic Function and Strain

**Figure 2:**
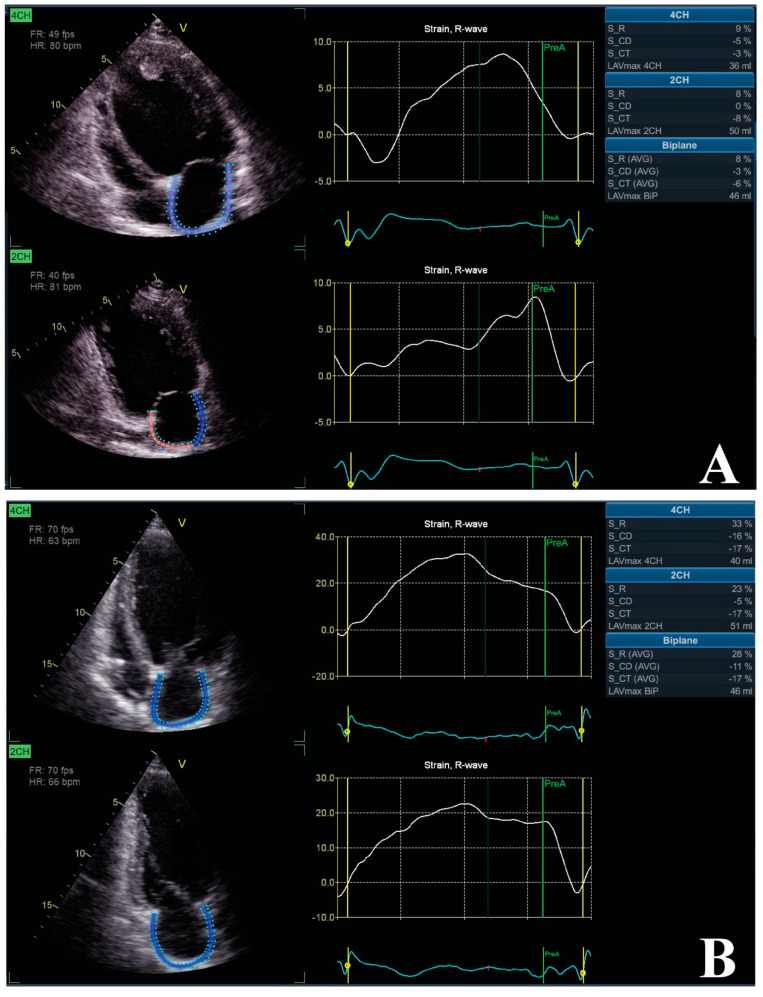
The impact of CRT on LA strain. Patient with dilated cardiomyopathy, LVEF of 33%, and QRS morphology of LBBB with duration of 153 msec. Echocardiographic evaluation using a General Electric VIVID E95 vendor and EchoPac software package to determine LA strains (Images from the personal collection of the authors). Initial evaluation showed impaired LA strains (**A**) and, after a 6-month follow-up, there is significant LA functional reverse remodeling with improved LA strains (**B**). Abbreviations: LA, left atrium; LBBB, left bundle branch block; LVEF, left ventricle ejection fraction.

More and more data support the relationship between CRT and LA phasic function and strain (Figure 2). The study by Donal et al. showed the positive impact of CRT on LA remodeling by improving LATF and LAS-r after implantation [93]. In the study by Badran et al., which sought to evaluate the interrelation between LA function and CRT, it was found that CRT responders had significant improvement in LA emptying fraction [89]. A recently published research paper aimed to evaluate the impact of CRT on LAS-r and LV global longitudinal strain, accounted as markers of cardiac reverse remodeling: incomplete reverse remodeling was considered as an improvement in LAS-r or LV global longitudinal strain, while the complete form was considered an improvement in both parameters. Therefore, the study concluded that improvement in both LAS-r and LV strain was independently associated with the lowest risk of mortality [94].

Moreover, the study by Dokuni et al. evaluated the impact of CRT on LA function. They proved that, at 6 months after CRT implantation, LAS-r significantly improved, while patients who had a LAS-r of over 14.6% at follow-up had considerably better prognosis as compared to others [95]. Moreover, another study followed to assess the predictive ability of LAS-r for LV reverse remodeling. They found that LAS-r was an independent predictor for positive response to CRT [96]. The study by Valzania et al. aimed to assess the changes in LA anatomical and functional remodeling at 12 months after CRT. At follow-up, they found that LAS-r was significantly improved in CRT responders and was correlated with improvement in mitral regurgitation, E/E’ ratio, LVEF, and LV global longitudinal strain, whereas in the CRT non-responders’ group, LAS-r worsened [97]. Likewise, in the study by Huntjens et al. conducted in patients who received CRT, increased values in LAS-r were correlated with event-free survival, while lower LAS-r was associated with MACEs [98].

Studies evaluating the link between LA remodeling and CRT are summarized in Table 2.

## 6. Future Perspectives

Emerging data report promising results regarding the link between CRT and LA remodeling, even though research is only in its infancy. Another important connector between CRT and LA function might be mitral regurgitation, due to its impact on LA size, shape, and function. It is known that CRT might become useful in managing functional mitral regurgitation due to the fact that cardiac dyssynchrony aggravates the severity of regurgitation [99]. Further studies should assess whether reducing mitral regurgitant volume and fraction by CRT might lower the burden on LA volumes and functioning and possibly improve LV diastolic dysfunction.

CRT is a useful minimal invasive procedure that restores synchronicity, along with the mitigation of LA dysfunction and remodeling. Patients should be rigorously selected in order to predict best responsiveness. Moreover, patients should be evaluated initially and at follow-up using the same vendors and same operators in order to ensure proper reproducibility.

On the other hand, LA remodeling parameters determined by echocardiography or CMR are closely related to CRT responsiveness. Nevertheless, limited data are still available, and more research should be conducted in order to accurately characterize LA remodeling in patients who require CRT. Furthermore, further studies might require evaluating the ability of these parameters in predicting accurate response to CRT and, also, if they might become useful day-to-day measurements that might add incremental value towards cardiac resynchronization, either by simply combining with other clinical or imaging parameters, or by generating risk scores that would use them as well.

## 7. Conclusions

By restoring cardiac synchrony, CRT has positive impact on LA remodeling. In patients who are CRT responders, it reduces LA volumes, and it improves LA phasic functions and strain. Conversely, increased LA volumes and impaired LA strain are able to predict CRT responsiveness and outcome in these patients.

## Figures and Tables

**Table 1:** Studies endorsing the utility of cardiac resynchronization therapy.

Clinical Trial	n	Year	NYHA Class	QRS Interval	LVEF	Follow-Up
Multisite stimulation in cardiomyopathy (MUSTIC)	67	2001	III	>150 ms	<35%	6 Mo
Multicenter inSync randomized clinical evaluation (MIRACLE)	453	2002	III, IV	>130 ms	<35%	>6 Mo
Pacing Therapies for Congestive Heart Failure (PATH-CHF)	42	2002	III, IV	>120 ms	<35%	>6 Mo
Multicenter InSync Implantable Cardioversion Defibrillation Randomized Clinical Evaluation (MIRACLE ICD)	639	2003	III, IV	>130 ms	<35%	6 Mo
Safety and Effectiveness of Cardiac Resynchronization Therapy with Defibrillation (CONTAK CD)	333	2003	III, IV	>120 ms	<35%	6 Mo
Multicenter InSync Implantable Cardioversion Defibrillation Randomized Clinical Evaluation II (MIRACLE ICD II)	186	2004	II	>130 ms	<35%	6 Mo
Pacing Therapies for Congestive Heart Failure II (PATH-CHF II)	89	2004	III, IV	>120 ms	<35%	6 Mo
Cardiac Resynchronization Heart Failure Study (CARE-HF)	813	2006	III, IV	>120 ms	<35%	29.4 Mo
Comparison of Medical Therapy, Pacing and Defibrillation in Heart Failure (COMPANION)	1520	2009	III, IV	>120 ms	<35%	14.4 Mo
Resynchronization reverses Remodeling in Systolic left ventricular dysfunction (REVERSE)	610	2013	I, II	>120 ms	<40%	12 Mo
Multicenter Automatic Defibrillator Implantation Trial–Cardiac Resynchronization Therapy (MADIT–CRT)	1820	2014	I, II	>130 ms	<30%	24 Mo
Resynchronization-Defibrillation for Ambulatory Heart Failure Trial (RAFT)	1798	2014	I, II	>130 ms	<30%	60 Mo

Abbreviations: LVEF, left ventricle ejection fraction; Mo, month; n, number of patients; NYHA, New York Heart Association.

**Table 2:** Studies which evaluate the relationship between CRT and LA remodeling.

Authors	Ref	Year	n	Data	Follow-Up	Findings
Donal et al.	[83]	2009	46	LAPF LAVmax LAS-r	6 Mo for the impact of CRT on LA remodeling	CRT significantly improved LAVmax, LAVmin, LAS-r, LAPF inducing LA reverse remodeling
Brenyo et al.	[77]	2011	1785	LAVmax	6 Mo for the risk to develop atrial tachyarrhythmias	Lower LAV is correlated with lower incidence of atrial tachyarrhythmias and lower outcome
Rossi et al.	[80]	2013	52	LAVmax LAVmin LAVpre-A	6 Mo for the ability of LAVmax to predict CRT responsiveness	Lower LAVmax, LAVmin, and LAVpre-A were significantly associated with higher rate of CRT response
Kuperstein et al.	[76]	2014	1785	LAVmax	6 Mo for all-cause mortality and HF	Higher LAVmax is associated with outcome Each 1% reduction in LAV = 4% reduction in hazard for HF/death
Feneon et al.	[86]	2015	79	LAS-r LA pre-ejection index	6 Mo to evaluate the impact of CRT on LA strain	LAS-r was a good predictor for CRT responsiveness, regardless of cardiac pathology
Valzania et al.	[87]	2016	30	LAS-r	12 Mo for the impact of CRT on LA strain and the relationship with some parameters	CRT responsiveness had beneficial impact on LA size and LAS-r. LV function, filling pressures, and mitral regurgitation significantly influenced LA remodeling
Badran et al.	[79]	2017	37	LAVmax LAVmin LAVpre-A; LATF LAS-r; LAS-cd	3 Mo for evaluating LA remodeling	LAVmax, LAVmin, LAS-r, LAS-cd, LATF were significantly improved in those who were responders to CRT
Lupu et al.	[82]	2018	28	LAVmax LAVmin LAVpre-A; LATF, LAPF, LAAF	3 Mo for the impact of CRT on LA volumes and phasic functions	CRT has beneficial effects on LAVmax, LAVmin, and LAVpre-A, even within first days of implant
Dokuni et al.	[85]	2020	90	LAS-r LAPF	6 Mo for the role of CRT on LA strain function	CRT reduced LA dyssynchrony, which in turn improved LAS-r and LAPF
Stassen et al.	[84]	2022	923	LAS-r GLS	6 Mo for the impact of CRT on both LA and LV remodeling	Patients who exerted both LA and LV remodeling had lowest mortality risk

Abbreviations: CRT, cardiac resynchronization therapy; GLS, left ventricle global longitudinal strain; LA, left atrium; LAAF, left atrial active booster pump fraction; LAPF, left atrial passive emptying fraction; LAS-r, left atrial reservoir strain; LATF, left atrial total emptying fraction; LAVmax, left atrial maximum volume; LAVmin, left atrial minimum volume; LAVpre-A, left atrial pre-atrial contraction volume; LV, left ventricle; Mo, months; n, number of patients.

## Data Availability

Not applicable.
